# Clinical insights into nanomedicine and biosafety: advanced therapeutic approaches for common urological cancers

**DOI:** 10.3389/fonc.2024.1438297

**Published:** 2024-08-13

**Authors:** Mohammad Reza Fattahi, Mansoureh Dehghani, Somayyeh Paknahad, Shafa Rahiminia, Deniz Zareie, Behzad Hoseini, Tahmineh Rajaee Oroomi, Hossein Motedayyen, Reza Arefnezhad

**Affiliations:** ^1^ Student Research Committee, School of Advanced Technologies in Medicine, Shahid Beheshti University of Medical Sciences, Tehran, Iran; ^2^ Reza Radiotherapy and Oncology Center (RROC), Mashhad, Iran; ^3^ Legal Medicine Organization, Tehran, Iran; ^4^ School of Medicine, Guilan University of Medical Sciences, Rasht, Iran; ^5^ School of Medicine, Shahroud University of Medical Sciences, Shahroud, Iran; ^6^ School of Medicine, Mazandaran University of Medical Sciences, Sari, Iran; ^7^ Islamic Azad Tehran University of Medical Sciences, Tehran, Iran; ^8^ Autoimmune Diseases Research Center, Kashan University of Medical Sciences, Kashan, Iran; ^9^ Coenzyme R Research Institute, Tehran, Iran; ^10^ Shiraz University of Medical Sciences, Shiraz, Iran

**Keywords:** nanomedicine, nanoparticle, urology, cancer, treatment, clinical, biosafety

## Abstract

Urological cancers including those of the prostate, bladder, and kidney, are prevalent and often lethal malignancies besides other less common ones like testicular and penile cancers. Current treatments have major limitations like side effects, recurrence, resistance, high costs, and poor quality of life. Nanotechnology offers promising solutions through enhanced diagnostic accuracy, targeted drug delivery, controlled release, and multimodal imaging. This review reflects clinical challenges and nanomedical advances across major urological cancers. In prostate cancer, nanoparticles improve delineation and radiosensitization in radiation therapy, enable fluorescent guidance in surgery, and enhance chemotherapy penetration in metastatic disease. Nanoparticles also overcome bladder permeability barriers to increase the residence time of intravesical therapy and chemotherapy agents. In renal cancer, nanocarriers potentiate tyrosine kinase inhibitors and immunotherapy while gene vectors and zinc oxide nanoparticles demonstrate antiproliferative effects. Across modalities, urological applications of nanomedicine include polymeric, liposomal, and metal nanoparticles for targeted therapy, prodrug delivery, photodynamic therapy, and thermal ablation. Biosafety assessments reveal favorable profiles but clinical translation remains limited, necessitating further trials. In conclusion, nanotechnology holds significant potential for earlier detection, precise intervention, and tailored treatment of urological malignancies, warranting expanded research to transform patient outcomes.

## Introduction

Urological malignancies arising in the kidney, bladder, and prostate are prevalent cancers with substantial morbidity and mortality (1). They stand out as prevalent and lethal malignancies within the genitourinary system, and current theranostic strategies for these cancers are deemed inadequate, marked by limitations in sensitivity, specificity, efficacy, safety, and overall quality of life (2).

The drawbacks of current treatments for urological cancers are multifaceted and depend on the type and stage of cancer, as well as the patient’s age, health status, and preferences. Some common drawbacks of current treatments include the high risk of acute and long -term side effects that negatively impact patient well-being. For example, radical surgical procedures may lead to chronic pain, nausea, fatigue, and sexual dysfunction. Similarly, radiation and chemotherapy often cause severe gastrointestinal adverse events and cognitive impairment. Such side effects can be disabling or even life-threatening in elderly patients with comorbidities (1, 3). Additionally, localized urological cancers often recur and progress despite initial treatment. In the metastatic setting, hormone, and chemotherapies provide transient control but eventually spawn lethal therapeutic resistance (4, 5).

From diagnosis to end-of-life care, patients experience cumulative adverse events ranging from acute catheterizations, stomas, and cytopenias to long-term cognitive changes and secondary malignancies. Exorbitant costs further compound the physical and emotional side effects.

Thus, there is an unmet need for new diagnostic and therapeutic paradigms tailored to the unique biology of genitourinary tumors and the sensibilities of affected patients (6, 7).

To overcome these drawbacks, there is a need for novel and personalized approaches to urological cancer management, such as immunotherapy, targeted therapy, precision medicine, and multidisciplinary care. Realizing these opportunities necessitates additional preclinical validations and ethical evaluations to translate nanomedicine into widespread urologic practice responsibly. With concerted efforts across scientific disciplines and institutional infrastructures, nano-enabled care stands to make substantive impacts on the early detection, risk-stratification, and tailored treatment of genitourinary cancers.

Nanotechnology, offering advantages in urological cancer theranostics like enhanced permeability, targeted delivery, controlled release, and multimodal imaging, has explored various nanoparticle (NP) types such as metallic, polymeric, liposomal, and carbon-based NPs. Gold NPs enable a rapid urinary test for bladder cancer (BC), while magnetic NPs are utilized in magnetic resonance imaging and hyperthermia therapy for prostate cancer (PCa). Additionally, polymeric NPs have shown efficacy in chemotherapy and gene therapy for BC (8). However, despite the promising potential of nanotechnology, it is essential to ensure the biosafety of novel treatments. Biosafety refers to the assessment and management of the potential risks of nanomaterials to human health and the environment. Some of the factors that affect the biosafety of nanomaterials include their size, shape, surface charge, chemical composition, biodegradability, biodistribution, and biocompatibility. Therefore, it is essential to conduct rigorous preclinical and clinical studies to evaluate the biosafety of nanomaterials before they can be translated into clinical practice (9, 10). In this review, we aim to provide a comprehensive and updated overview of the role of nanotechnology in urological cancer treatment and the biosafety of previous studies, focusing on clinical relevance and insight. We also will discuss the current challenges and opportunities of nanotechnology in urological cancer, and highlight the future directions and perspectives of this promising field.

### Prostate cancer

In 2020, approximately 1.4 million cases of PCa were reported globally, making it the second most frequently identified cancer among men (11). The number of deaths due to PCa in 2020 was 375,000 worldwide. According to the World Health Organization (WHO), PCa is the most often diagnosed male cancer in 112 countries and the leading cancer-related death in 48 countries. Moreover, corresponding socio-economic burden is enormous; PCa treatment costs increase more rapidly than for any other cancer (12).

#### Current treatment options and their limitations

Localized PCa has various treatment options resulting in controversy among experts and confusion leading to frustration and stress in patients (13).

Surgical prostatectomy is an important treatment option for all risk groups of localized disease and even for selected patients with regional nodal metastasis. Although surgical indications become more limited as medical treatment options for PCa continue to expand, radical prostatectomy remains a crucial option in many patients (14). The improvement of anatomic knowledge and technical methods as well as the advent of laparoscopy and robotic-assisted surgery, have changed the historically morbid and lethal prostatectomy to a rather safe procedure becoming a common treatment approach for localized PCa (15, 16); However, this treatment method is still invasive and faces the risk of complications such as blood loss, postoperative pain, surrounding structures injury, reoperation, infection, urine leak, incontinency, long-time catheterization, anastomotic stricture, thromboembolism, heart attack, and death (16). In addition, there is still no consensus about some technical decisions such as whether to dissect lymphatics or not (17).

Radiation therapy has also undergone significant advancements as the pivotal treatment modality for most cases of local and locally advanced PCa. There is a remarkable increase in the utilization of highly conformal intensity-modulated radiation therapy (IMRT), and image-guided radiation therapy (IGRT), which implements fiducial markers placed in the targeted tissue for daily localization, and stereotactic external beam radiation therapy, irradiating small target volumes with high doses of radiation in few fractions (13). Interstitial brachytherapy is also widely used alone or as an additional radiation boost for localized PCa patients (18).

Although all these methods take effective steps towards increasing the radiation dose to the target and saving healthy organs which is particularly important, when it comes to cancers that occur in the complex anatomical site of the pelvis, various acute and chronic side effects of radiation therapy continue to bother PCa patients, especially in the first four years after treatment (13). There is a wide range of radiation side-effects including acute complications caused by inflammation such as cystitis, urethritis, and proctitis manifesting as frequency, urgency, and discomfort during urination or bowel movements (19). Such side effects force patients to make strict dietary changes and take more medications to control side effects (20). On the other hand, there are long-term complications, like various disorders related to sexual and digestive functions and probable strictures and obstructions, which, despite having a greater impact on the quality of life of patients, are more neglected than acute complications (21, 22).

Other than medical castration and local palliative treatments, systemic treatment for metastatic PCa, specifically metastatic Castration-Resistant Prostate Cancer (mCRPC), includes a few chemotherapeutic options, targeted therapy, and immunotherapy (23). Chemotherapy leads to multiple acute and chronic side effects from gastrointestinal discomfort and hair/skin conditions to serious toxicities such as cytopenia, immune suppression, leukemia, and severe hypersensitivity reactions (24). On many occasions, the severity of toxicities results in dose modification and treatment discontinuation. Immunotherapeutic drugs that engage the patient’s immune system in the battle against malignant cells have drawn enormous attention recently. Although promising in multiple malignancies, immunotherapy provides few options with currently less than significant results for PCa, including programmed cell death protein 1 (PD-1) and cytotoxic T-lymphocyte antigen 4 (CTLA-4) blockage and vaccine-based pharmaceuticals (25, 26). While investigations on the role of immunotherapy in metastatic PCa continue to evolve, concerns are raised regarding a wide and not fully understood range of treatment-related toxicities due to the close connection of the immune system with various organ systems and random events of systemic immune activation caused by the intravenous systemic drug administration (27).

Nuclear medicine and bone-seeking radiolabeled pharmaceuticals have demonstrated encouraging results in metastatic PCa treatment, particularly since the discovery of the prostate-specific membrane antigen (PSMA) protein as a beneficial diagnostic and therapeutic target (28); However, multiple adverse effects, including anemia, changes in blood parameters, liver enzyme elevation, nephrotoxicity, fatigue, nausea, and dry mouth, are reported in a considerable proportion of patients (20).

Androgen deprivation therapy (ADT) is a form of hormonal intervention used as a major component in the treatment of PCa across all risk groups (23). PCa’s proliferation is testosterone dependent whether it is local or metastatic and suppression of the androgen-signaling axis is a key ingredient in PCa management recipe. One routine option that is still recommended in individual metastatic patients is the surgical removal of testicles (orchiectomy); However, medical castration with Luteinizing Hormone-releasing Hormone (LHRH) agonists and antagonists, antiandrogens, and novel enzymatic steroidogenesis inhibitors is the preferred option with manageable duration (19). Castration, regardless of whether it is surgical or medical, often leads to various adverse effects that can be a source of frustration and challenges for patients. These side effects include sexual problems, hot flashes, metabolic disorders, muscle mass decline, sleep disturbances, mental health issues, genitourinary difficulties, bone loss, weight gain, and anemia, all of which can significantly affect the patient’s quality of life. Whether surgical or medical, castration results in significant toxicity which is the major source of challenge and frustration in most cases. These adverse effects include a long list of highly prevalent effects such as sexual problems, hot flashes, metabolic disorders, muscle mass decline, sleep disturbances, mental health issues, genitourinary difficulties, bone loss, weight gain, and anemia, all of which dramatically affects the patient’s quality of life. On the other hand, castration resistance, meaning disease recurrence or progression while sufficient testosterone suppression is evidenced, eventually occurs in all patients treated with ADT (29). This will leave the patient with limited more toxic treatment options such as chemotherapy and immunotherapy. Increasing the accuracy of drug delivery to the cellular level can prevent further development of drug resistance that deprives the patient of the remaining options (30).

Delivery of drugs to malignant cells can significantly reduce side effects in different scenarios of PCa, such as localized, metastatic, or castration resistance. This is the primary topic of discussion in nanomedicine.

#### Nanotechnology applications in PCa therapy

Nanotechnology has a meaningful role in urological cancers, especially PCa in the field of prevention, diagnosis, and treatment. It can address multiple aspects of treatment drawbacks mentioned above, in nearly every treatment modality. Better chemotherapy drug delivery can cause a higher rate of remission. There are a variety of uses like magnetic, lipid-based, and gold NPs; Progress in imaging can facilitate the diagnosis and NPs can enhance water solubility and accumulation in targeted cells for chemotherapy drugs which causes better therapeutic outcomes (31).

In surgery, the accumulation of nanoparticles in the diseased area, specifically metal nanoparticles such as Gold, can increase the detection accuracy before and during the operation (32, 33). Moreover, the nanoparticle can be further improved via conjugation to specific ligands targeting antigens like PSMA (34), or formulation with ablative agents to perform the treatment on its own (35, 36). In a recent study, Wu et al. developed a Cy-KUE-OA nanoprobe to facilitate PCa surgery via fluorescence guidance. They utilized Glutamate-urea-lysine (KUE) as a promising PSMA ligand and Oleic acid (OA), a monounsaturated fatty acid that is actively accumulated in malignant cells, to deliver a cyanine-based fluorescent dye which is activated after both uptake and infrared radiation (37). This methodology can be performed with various ligand types, vehicles and active substances, taking benefit from the flexibility and structural versatility. Teh et al. incorporated nanoprobe technology in two modalities (38). They reported successful implementation of targeted nano-probes for intraoperative visualization of tumoral cells and postoperative targeted drug delivery in mice models and proposed the potential of this method to improve multimodality treatment in clinical studies.

In radiation therapy, remarkable advances in technology, accuracy and variety of modalities of radiotherapy devices in the last twenty years have coincided with the emergence and expansion of the use of nanotechnology in medicine (39). With the spread of modern complex radiotherapy techniques using highly non-uniform radiation intensity to deliver elegant conformal dose distributions that are especially useful in the treatment of pelvic tumors such as prostate, accurate diagnosis of the tumor area has become much more important comparing to previous 2D and 3D techniques (13). Precise delineation of the tumor is the first key to an optimal treatment planning and radiation delivery resulting in high tumor dose (more than 80 Gy to the planning target volume [PTV] in IMRT) and less organ-at-risk radiation (40, 41). This crucial factor can be improved using fluorescent or metal nanoparticles targeting and accumulating in the malignant cells. Current imaging techniques used for tumor delineation discriminate the tumoral tissue based on differences in contrast, functional attenuation, or magnetic resonance, whilst nanoparticles can detect the malignancy on cellular level. On the other hand, the particles can also act as targeted radiosensitizers (42, 43). Moeendarbari et al. took a step further and incorporated the radioisotope palladium-103 in gold nanoparticles to develop an internal radiation treatment for unresectable tumors and reported a dramatic response of more than 80% tumor shrinkage in PCa xenograft models (44). Currently, ongoing clinical trials are investigating various applications of nanomedicine in treatment with ionizing radiation; However, modern paradigms such as tumor microenvironment and immunogenicity must be taken into consideration before the adoption of these methods into patient treatment (42).

Numerous systemic therapeutics can become incorporated in nanostructures to enhance the effect, target specific cells, and save normal organ-systems (45). Nanomedicine drug delivery systems can increase drug solubility, extend half-life, and regulate drug release by adjusting their chemical and physical properties such as hydrophilicity, charge, size, and the nature of their surface ligands. Moreover, nanomedical drug conjugates can penetrate target cells in PCa via mechanisms mediated by receptors, making them the front line of anti-cancer drug experiments (46). These features become specifically beneficial regarding castration resistant PCa, in which the only effective options left are a number of systemic treatments, the maximum efficiency of which and avoiding discontinuation of treatment due to complications is critical (47). Most PCa deaths are caused by Castration-Resistant Prostate Cancer (CRPC), and CRPC patients are at high risk of developing drug resistance. Previous studies have developed new nanoparticles called lipid-calcium-phosphate arginine-glycine-aspartic acid (LCP-RGD) NPs that can deliver small interfering RNA (siRNA) and Docetaxel (DTX), resulting in significant therapeutic effects. By encapsulating the chemotherapeutic agent, with dioleoyl phosphatidic acid (DOPA) and distearoyl phosphatidylethanolamine-polyethylene glycol (DSPE-PEG), the calcium phosphate core of the NPs can efficiently aggregate the negatively charged siRNA. The synergistic effects of DTX and GRP78 siRNA are associated with changes in the cell cycle, autophagy, and apoptosis following GRP78 silencing, which can enhance the destruction of tumor cells by DTX (47).

In addition to the role of nanomedicine in the conventional treatments mentioned above, a wide range of herbal and complementary treatments can be formulated in nanoparticles, alone or in combination with chemotherapy or immunotherapy, as reviewed in detail by Cherian et al. (48). These complementary medications can further enhance the treatment efficacy or reduce side-effects, without changing the original method, treatment time, or number of administrations. Additional encouraging uses of nanomedicine in the treatment of PCa including gene therapy via delivering mRNA structured with antiproliferative and apoptotic effects, photodynamic therapy using photosensitizing agents, and immunotherapy (**Table 1**).

**Table 1:** Inventory of nanomedicines used to treat prostate cancer and their biosafety.

Cancer Type	Treatment strategies	Nanoparticles	Clinical Effect	Biosafety	Reference
Prostate Cancer	Drug delivery	CUR NP	Restore the potency of CUR in both resistant DU145 cells and PC3 cells	No cytotoxicity	(49)
	Targeted therapy	AgNP-PLE	This substance induces cell cycle arrest and triggers apoptosis in human prostate cancer cells	Less toxic to normal cells	(50)
	Targeted therapy	RSV-SLN	As potential carriers for drug delivery of chemotherapeutics at an extended systemic circulation and targeting efficiency at the tumor site	Good biosafety	(51)
	Immunotherapy	MGF-AuNP	M2-type macrophages that are polarized have been found to improve the immune response	No toxicity to normal cells	(52)
	Aminolysis	PHB-PEI NP	Excellent biocompatibility and high transfection efficiency for cancer therapy	No significant cytotoxicity effect	(53)
	Targeted therapy	PTX/siRNA NP-Apt	Superior efficacy was achieved with minimal side effects in the subcutaneous and orthotopic PCa tumor model with enhanced tumor-targeting ability	Reduce toxic	(54)
	Drug delivery	LNP-shPKN3	This lipid nanoparticles treatment had a high rate of tumor suppression at 65.8%	Lower toxicity	(55)
	PDT, PTT	ICGNP	Enhance photothermal therapy and decrease in cell viability was mainly the result of photothermal action	No toxicity	(56)
	PDT	PEG-b-PNBMA	Nano assemblies of PEG-b-PNBMA loaded with Rose Bengal lactone act as a smart nanocarrier for photosensitizer delivery. Sequential ‘405-580 nm’ irradiation on RB-M treated 22RV1 cells produced the best PDT outcome.	No obvious toxicity (By MTT assay)	(57)
	Immunotherapy	mRNA vaccine NP	Increasing the tumor-associated antigen presentation, also promoting CD8 T cell recruitment into the tumor, and enhancing the overall anti-tumor response	Minimal or no cytotoxic effect	(58)
	Immunotherapy	RALA/pDNA NP	Induced a tumor-specific cellular immune response, and inhibited the growth of TRAMP-C1 prostate tumors in both prophylactic and therapeutic challenge models *in vivo*	Undetermined toxicity (By MTT test)	(59)
	Drug delivery		Codelivery of DTXL and GRP78 siRNA enhances the anti-prostate cancer effects *in vitro* and *in vivo* and sensitizes the cell-killing effect of DTXL. This method may be especially useful for treating drug-resistant CRPC	No systemic toxicity (By MTT test)	(47)
	Targeted therapy	GNP + DTX	*In vivo*, GNP/DTX/RT treatment showed significant tumor growth reduction and 100% mice survival compared to other conditions	Reduction in some of the normal toxicities associated	(60)
	Chemo-phototherapy (Dual function)	CuSNP	Efficient drug release, effective cytotoxic activity, and maximum tumor growth inhibition during *in vivo* studies	Low systemic toxicity and better biocompatibility	(61)
	Drug delivery	Tf-CRC-SLNs	*In vivo* studies on mice with PCa demonstrated significant tumor regression, indicating the potential of bioconjugated SLNs for cancer treatment	Minimal cytotoxicity	(62)
	Drug delivery	Gen@AuNP	Inhibited the growth of three prostate carcinoma cell lines and were selective towards malignant phenotype	Low toxicity (By MTT test)	(63)
	Drug delivery & Combination therapy	@PCEC NP	DOX-EZ-loaded NPs were more cytotoxic to PC3 cells than single drug-loaded NPs, indicating a synergistic effect	Negligible cytotoxicity on the PC3 cell line (By MTT test)	(64)
	Drug delivery	NLPs-RGD-Cur-ATO	This co-delivery enhanced anti-proliferative effect, increased apoptotic cells, and reduced EGFR gene expression level	Decrease cytotoxicity (By MTT test)	(65)
	Gene therapy	Apt-PEG-siRNA@ZIF-8 NP	Aptameric targeted therapy of SNHG15 siRNA to prostate cancer cells in both cell lines and xenograft mouse model demonstrated significant antiproliferative and apoptotic effect on malignant PC cells.	No side effects observed in vital organs	(66)
	Tumor-targeted imaging agents	Cy-KUE-OA- PSMA	This study introduces a targeted NIR probe for PCa that accurately removes tumors using fluorescence-guided surgery with high sensitivity and selectivity	Low cytotoxicity (By MTS assay)	(37)
	Tumor targeting & Radiotherapy	AuNP	PSMA-targeted gold nanoparticles (AuNPs) are effective radiosensitizers for PCa, which demonstrated dramatically higher uptake and significantly improved radiotherapy efficacy	Potential liver toxicity	(43)
	Radiotherapy	AuNP	Human PCa cells were sensitized to 6-Megavolt X-ray radiation damage via targeted nanoparticles (AuNPs) and Radiobiological mitochondrial damage was significantly higher	Not toxic (By MTS assay)	(67)
	Radiotherapy	PSMA-AuNP	The radio sensitizing effect of PSMA-bound gold NPs in clinical megavoltage treatment was quantitatively measured via micro-dosimetry	No toxicity towards LNCaP cells	(68)
	Radiotherapy	Pd-Au Nano seeds	Radioactive nano seeds effectively treated xenograft models of PCa and shoed more than 80% tumor size reduction	No noticeable liver or kidney toxicity observed	(44)
	Drug delivery	Poly TTG-SS@DTX NP	DTX-loaded poly-Tetra ethylene glycol NPs rapidly release the chemotherapy drug in tumor microenvironment *in vitro* and spare normal cells and the killing effect of it on C4-2 cells was stronger than free anti-tumor or free DTX combined with the blank nano-carrier	Good compatibility with healthy cells (By MTT assay)	(69)
	Photo-immunotherapy	YBS-BMS NP-RKC	Near-infrared-activated polymer photosensitizer YBS and PD-1/PD-L1 complex inhibitor BMS-202 were encapsulated in a targeted NP to treat PCa *in vivo* and *in vitro*. This therapy generates a strong immune response against tumors, halting primary tumor progression and preventing tumor relapse and metastasis through long-term immune memory.	Low toxicity	(70)
	Multimodality treatment	Au-TNF NP	AuNPs were conjugated with Tumor Necrosing Factor enhanced vascular permeability (80%) and restricted tumor growth (60%)	Systemic toxicity, especially muscles	(71)

**Figure 1:**
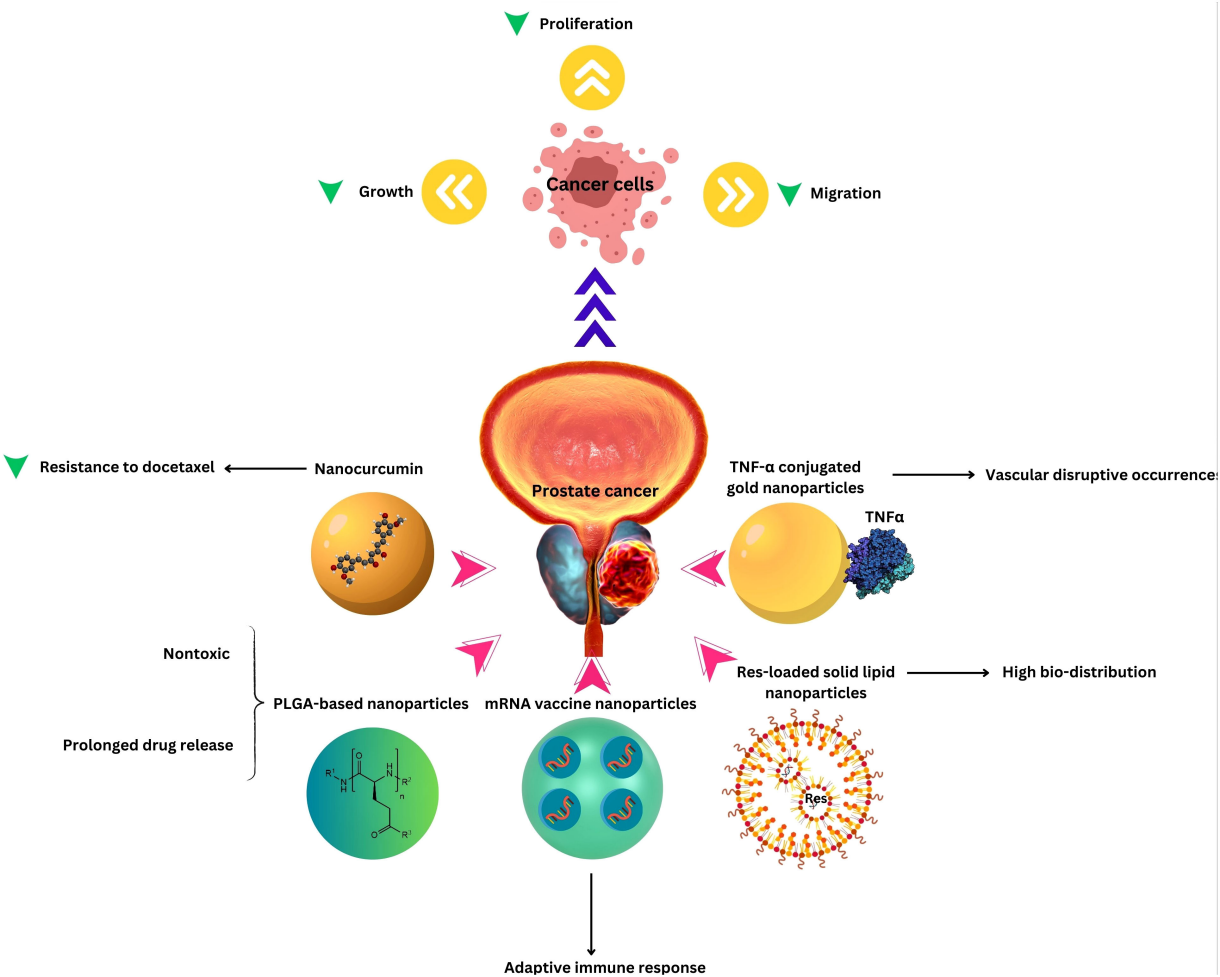
Nanotechnology applications in Prostate Cancer Therapy.

Nanotechnology’s impact on PCa treatment extends to advanced targeted therapy and image-guided interventions. Through the use of nanocarriers, therapeutic agents can be precisely delivered to PCa cells, enhancing treatment specificity. Targeted therapy at the molecular level allows for tailored approaches, interfering with specific signaling pathways involved in cancer growth (72, 73). Regardless of the type of treatment, the prognosis varies and is influenced by many factors, but all treatments can affect sexual function and quality of life to different extents. The integration of nanotechnology in targeted therapy and image-guided interventions represents a significant advancement in the field of PCa treatment. By combining therapeutic precision with real-time monitoring, nanotechnology holds the potential to improve treatment outcomes, minimize side effects, and offer a more personalized and effective approach to managing PCa (**Table 1** and **Figure 1**).

### Bladder cancer

In 2020, bladder cancer (BC) ranked as the tenth most frequently identified cancer globally, with an estimated 573,000 new cases and 213,000 deaths attributed to the disease (74). The American Urological Association (AUA) provides guidelines for classifying non-muscle invasive bladder cancer (NMIBC) into high-, intermediate-, and low-risk groups, which helps in tailoring treatment and surveillance strategies; both carry significant morbidity rates (75, 76). Despite advancements in treatment, NMIBC is notorious for its high recurrence rates, which can range from 15% to 61% within the first year and between 31% and 78% within five years. The economic impact of managing and treating bladder cancer which is increasing globally is substantial, with costs associated with diagnosis, treatment, and follow-up care contributing to a significant financial burden on healthcare systems (77).

#### Current treatment options and their limitations

Intravesical therapy has become a mainstay in the management of NMIBC. This approach involves the direct instillation of therapeutic agents into the bladder, targeting residual malignant cells following transurethral resection. The agents used for intravesical therapy include Bacillus Calmette-Guérin (BCG), mitomycin C, and gemcitabine (78). These agents can be used alone or in combination to induce cytotoxic effects or stimulate an immune response against cancer cells. However, the effectiveness of intravesical therapy is often hampered by the bladder’s natural permeability barrier, which limits drug penetration and reduces the duration of drug residence in the bladder, potentially leading to treatment failure (79, 80). Although intravesical treatment is widely utilized as the main tool to prevent recurrence in superficially resected BC, current management of NMIBC is far from sufficient. One-year recurrence rates and 5-year recurrence rates are reported in a range of 15–61% and 31–78% respectively (81). The bladder permeability barrier (BPB) on the urothelial surface decreases substance penetration and the repeating emptying of the drug and its dilution by urine leads to short residence time, unsustainability, low penetration, and failure to release with consistency (82).

To address these challenges, researchers are exploring innovative strategies to enhance the delivery and efficacy of intravesical therapy. These include the development of nanocarriers that can improve drug solubility and retention, magnetic particles that can be directed to tumor sites, and gene therapy approaches that target specific molecular pathways involved in bladder cancer progression (78, 83, 84).

For MIBC, treatment typically involves more aggressive interventions, with radical cystectomy being the standard of care. This surgical procedure entails the complete removal of the bladder and surrounding organs, including the prostate in males and, in females, the anterior exenteration of the bladder, urethra, vaginal wall, and uterus. While radical cystectomy is effective, it is also associated with significant morbidity and impacts on quality of life. Partial cystectomy is rarely feasible and there is often the need for neobladder construction via reconfiguring a portion of the intestine (85, 86).

Although robotic-assisted and laparoscopic surgeries, which are increasingly being adopted in clinical practice, offer less invasive alternatives with the potential for reduced complications and quicker recovery times (87), the invasive nature of the optimal therapeutic surgery still leads to various short- and long-term complications including multiple metabolic and neuromechanical side effects. Replacing the bladder with an absorptive structure results in numerous electrolyte imbalances and vitamin deficiencies which then cause dysfunction in other organ systems (85), as well as long-term complications such as obstructions and strictures. Tumor recurrence is another common consequence in some studies, 86% of which developed within the first 3 years of cystectomy (88). While certain individuals may derive advantages from the seemingly comparable option of bladder-conserving three-modality therapy, involving transurethral resection followed by chemoradiotherapy, this approach comes with its own array of negative outcomes (89), which can be mitigated to a considerable degree through the utilization of nanomedical drug delivery methods.

Radiation therapy, chemotherapy, and immunotherapy serve as adjunctive treatment modalities for both NMIBC and MIBC cases. Local resection along with radiation and chemotherapy is also an option for local MIBC cases who are not selected for radical therapy or have negative features in the pathologic assessment of the surgical tissue (80, 86). However, each modality presents its own set of challenges. Radiation therapy, despite the use of modern techniques such as IMRT and IGRT, may lead to genitourinary and gastrointestinal dysfunction due to challenges in adequately targeting and sparing normal tissues (90). Although chemotherapy and immunotherapy are novel treatment options that have shown promise in clinical trials, they are not yet widely available and have numerous side effects that are neither completely investigated in research nor sufficiently familiar in the clinic (27).

The economic impact of bladder cancer is substantial, with management costs being among the highest for any malignancy. Recurrent disease, the need for ongoing surveillance, and the introduction of novel therapies all contribute to the financial burden of bladder cancer care. In the European Union, bladder cancer treatment accounts for approximately 3% of all cancer costs, representing a significant healthcare expenditure (91).

Enhancements in the biological comprehension of bladder cancer have revealed new potential targets for BC treatment strategies. Among these advancements are immunotherapy, targeted therapy, and antibody-drug conjugates, all of which have demonstrated potential in bettering patient outcomes with bladder cancer (92).

#### Nanotechnology applications in BC therapy

The clinical management of bladder cancer presents a complex array of challenges. A key issue is the high propensity for tumor recurrence and progression following initial treatment modalities. This complication arises in part from the limitations inherent to current diagnostic techniques, which may fail to accurately detect and characterize the disease state. The ability to reliably distinguish between NMIBC and MIBC is crucial, as these subtypes warrant distinct therapeutic approaches and clinical monitoring strategies. Consequently, there remains a pressing need for enhancements to the existing paradigms governing bladder cancer diagnosis, treatment selection, and post-treatment surveillance in order to improve overall patient outcomes and mitigate the substantial socioeconomic burden imposed by this malignancy (79).

Adhering to a strong variety of uroplakins and transmembrane proteins, the bladder mucosal layer poses a significant barrier to the effective entry of therapeutic medicines into neoplastic tumors (93, 94). Overcoming this biological impedance necessitates innovative therapeutic strategies. In this regard, the burgeoning fields of nanomedicine and biomaterials engineering have made notable strides, holding considerable potential for facilitating targeted intravesical delivery of anticancer agents; a group of researchers, for example, fabricated a poly-(L)-glutamic acid-based nanoparticle system to deliver drugs specifically to bladder cancer cells via intravesical administration. They utilized the lectin WGA as a targeting ligand to facilitate binding and internalization of the nanoparticles by bladder tumor cells, thereby enhancing therapeutic efficacy while overcoming the barrier imposed by the bladder mucosal lining. These cutting-edge approaches may not only enhance drug bioavailability at the tumor site, but could also synergize with and augment the efficacy of existing treatment modalities (95). Nanomedicine and biomaterial-based drug delivery platforms offer innovative approaches to enhance bladder cancer treatment outcomes (96). The unique advantages offered by nanotechnology in cancer treatment applications include improved drug efficacy and/or reduced toxicity, spatial and temporal drug release, enhanced drug stability, solubility, and tumor site accumulation, facilitated delivery of polymeric drugs to intracellular sites of action, codelivery of multiple drugs or therapeutic modalities for combination therapy, improved diffusion of drugs across tight epithelial and endothelial barriers, and sensitive imaging and accurate diagnosis with visible drug delivery or real-time feedback on the *in vivo* efficacy of a therapeutic agent, advantages that have been described in previous reviews (31, 94).

Expanding the purview beyond this, an investigation demonstrated that nano contrast agents used in conjunction with MRI, proffer a non-invasive modality for monitoring prognosis and therapeutic responses among patients afflicted with bladder cancer, furnishing clinicians with invaluable insights into disease trajectory and therapeutic efficacy (97). Photothermal nanoprobes (PNPs) represent an epochal innovation, endowed with the capacity to discriminate cancerous cells through thermogenic or fluorescent mechanisms under infrared illumination. This pioneering approach portends unprecedented potential for early detection, precise localization, and targeted therapeutic interventions, heralding a paradigm shift in bladder cancer diagnostics and therapeutics. Moreover, PNPs loaded with DOX have exhibited a protracted half-life subsequent to intravenous administration, eclipsing the efficacy of DOX in isolation and potentially obviating the exigency for recurrent dosing, thereby engendering diminished patient burden and heightened treatment compliance (98).

In advancing nanomedicine for bladder cancer, biosafety considerations are paramount. Ensuring the biocompatibility and safety of nanotechnological interventions is imperative for their translation into clinical practice. While nanomedicine holds immense promise, addressing concerns regarding toxicity, immunogenicity, and long-term effects is crucial for its widespread adoption. Rigorous preclinical evaluation and regulatory oversight are essential to ascertain the safety profile of nanomedicine techniques, fostering confidence in their clinical utility.

**Figure 2:**
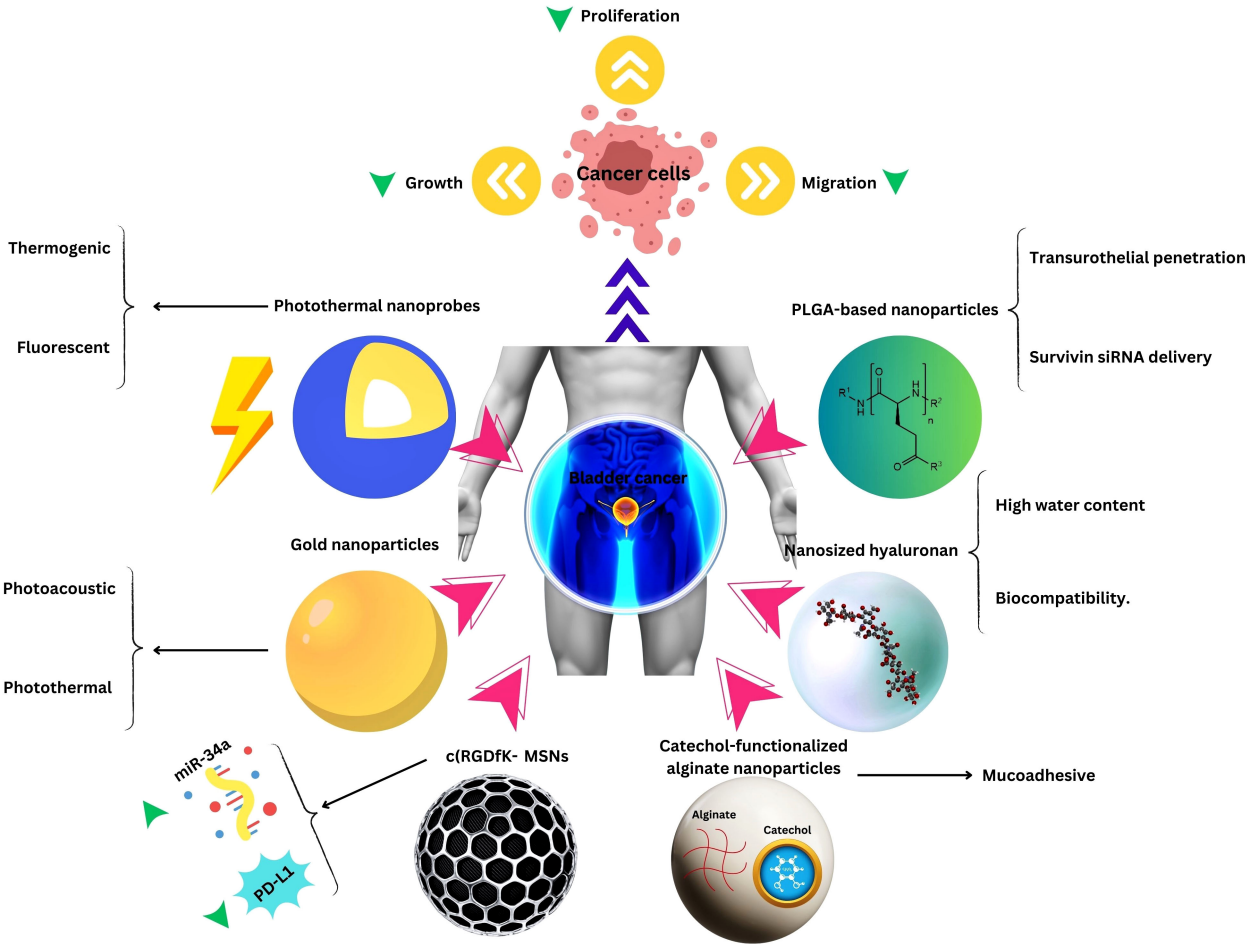
Nanotechnology applications in Bladder Cancer Therapy.

In **Tables 1**–**3**, we review recent nanomedical approaches in bladder cancer as well as other common urological cancers, encompassing polymeric NPs, lipid NPs, metallic NPs, and their applications in drug delivery, imaging, and therapeutic interventions. These advancements exemplify the potential of nanotechnology in revolutionizing bladder cancer management (**Figure 2**).

**Table 2:** Inventory of nanomedicines used to treat Bladder cancer and their biosafety.

Cancer Type	Treatment strategies	Nanoparticles	Clinical Effect	Biosafety	Reference
Bladder Cancer	Targeted therapy	Paclitaxel gelatin NP	Drug dilution by newly produced urine can be overcome, reducing treatment frequency while maintaining sustained drug levels in tumors	Unlikely any systemic toxicity	(99)
	Targeted therapy	HA/CHI nanoparticle-aggregated HET	HA nanoparticle aggregation strengthened the cytotoxic, antimigratory, and apoptosis-inducing activities against bladder carcinoma cells while reducing the viability-inhibitory effects on normal fibroblasts	Reduce the toxicity on normal cells (By MTT test)	(100)
	Drug delivery	Cat-Alg NP	These NPs have the potential to serve as a mucoadhesive drug delivery system for the treatment of bladder cancer	Not cytotoxic to MB49 cells	(101)
	PDT	Au@TNA NP	Enhance the cytotoxicity of PDT to cancer cells while minimizing toxicity to normal cells	Very low cytotoxicity (By MTT test)	(102)
	PDT	PLZ4 NP	Generate ROS and induce protein carbonylation and dendritic cell maturation	Standard cytotoxic chemotherapy	(103)
	Immunotherapy	AB680@EMVs-aPDL1	Provided adequate biosafety, and enhanced tumor targeting in a mouse model of bladder cancer	Adequate biosafety	(104)
	Immunotherapy	GNP-LLO91–99	Reduced tumor burden 4.7-fold and stimulated systemic Th1-type immune responses	No cytotoxicity	(105)
	Drug delivery	Modified MSN MSNP	This Nano-carrier (Mesoporous silica NPs) reduces bladder tumor growth by releasing miRNAs and siRNAs, which reduces CD44 expression, proliferation, migration, and invasion with minimal side effects	No cytotoxicity on cells	(106)
	Permeability enhancers	Nano papain	Papain enzyme improved drug permeability by 2-fold and reduced tissue penetration time to 0.6 hours.	Uncertain biosafety	(107)
	Immune PTT	SYMPHONY	This Plasmonic gold nano star (GNS) treatment induced long-lasting immunity against MB49 cancer cells	No signs of acute toxicity	(108)
	Targeted therapy	SERS NP	Passively targeted nanoparticles can penetrate deeper and bind to tumor tissue at higher concentrations in cancer than normal bladder urothelium.	Unknown toxicity profile	(109)
	Chemo-photothermal therapy	CS/PNIPAAm@SWCNTs	NIR-induced hyperthermia dilates tumor blood vessels, leading to superior tumor-targeting of nanomedicines compared to passive-targeting nanomedicines.	Minimized toxicity	(110)
	Targeted therapy	AuNP	AuNPs were found to be concentrated in the stroma surrounding tumor cells in mice with muscle-invasive BC, showing potential for enhancing radiotherapy	No long-term toxic	(111)
	Drug delivery	HG-MNS-DOX	Improved ability to treat cells with an RF field, resulting in over 95% cell death within 24 hours due to synergistic behavior	No cytotoxic effect	(112)
	mRNA delivery	KDM6A-mRNA	This approach demonstrates KDM6A’s therapeutic potential in inhibiting the metastasis of BC	Prevents potential toxicity	(113)
	PTT	Au–Ag@PDA NP	Inhibited T24 cell growth, altered cell cycle distribution, induced apoptosis, and triggered autophagy upon laser irradiation	Low cytotoxicity	(114)
	PTT	FePPy-NH2 NP	Used for MRI, photoacoustic imaging (PAI), and PTT, with a high photothermal conversion efficiency of about 44%	Low cytotoxicity	(115)
	Imaging and targeted chemotherapy	UCNP-AuNR	UCNP-AuNR nanoclusters are functionalized with EGFR antibodies to target bladder cancer cells that overexpress EGFRs	No cytotoxic effect	(116)
	Drug delivery	Fe3O4-MNP	Magnetic injectable hydrogels prolonged BCG residence time and induced a stronger immune response and higher antitumor efficacy than traditional BCG therapy for superficial BC	Bioerosion are nontoxic	(117)
	Immunotherapy	BCG-CWS NP	The coadministration of CWS-NP and ovalbumin (OVA) loaded NP resulted in the generation of OVA-specific cytotoxic T cells and inhibited the growth of E.G7-OVA tumors	No significant systemic toxicity	(118)
	Drug delivery	BCG-CWS	*In vivo*, antitumor efficacy studies revealed that the BCG-CWS-loaded liposomes effectively inhibited tumor growth in mice bearing MBT2 tumors.	Selective toxicity	(119)
	PTT	FCS- Cu2-xSe NP	Efficient transmucosal delivery and high penetration improve PTT effect both *in vitro* and *in vivo*	No appreciable toxic	(120)
	PDT	TiO2	The photocatalyst boosts photo response in visible and near-infrared regions, generating reactive oxygen species (ROS) that can kill cells when exposed to 808 nm light	Low toxicity	(121)
	Drug delivery	CS/PEG NP & CS NP	Both free I3C and I3C-loaded NPs significantly reduced T24 cells viability in concentrations ranging from 500 to 2000 μM after 24 hours of exposure	Cytotoxic effect on T24 cell line (By MTT test)	(122)
	Drug delivery	PTX/CS NSs	High capacity for loading drugs and maintain the sustained release of paclitaxel for over ten days	Low toxic	(123)

**Table 3:** Inventory of nanomedicines used to treat Kidney cancer and their biosafety.

Cancer Type	Treatment strategies	Nanoparticles	Clinical Effect	Biosafety	Reference
Kidney Cancer	Targeted therapy	Sor-Mag-SLNs	Enhances drug delivery to tumors while reducing damage to normal tissues	Reduced systemic toxicity	(124)
	Targeted therapy	Resveratrol NP	Inhibition of RCC cell migration and invasion through regulation of MMP2 expression and the ERK pathway	Reducing toxic (By MTT test)	(125)
	PTT	HSA-AuNR-TKI	When irradiation is paired with gold particles and drug-loaded NPs, the combined therapy showed the most significant and synergistic complete tumor necrosis of 100%	Reduced damage to surrounding cells	(126)
	Immunotherapy	TLR7/8 agonists encapsulated in PLGA NP	Trigger a robust antigen-specific immune response and are highly effective as vaccine adjuvants for cancer immunotherapy	Good biosafety	(127)
	Immunotherapy	CA IX-C4.16 NP	Combination of CA IX-C4.16 with Sor showed targeted delivery of payload in hypoxic tumors, resulting in induction of multimodal anticancer effects, including the resurrection of apoptosis, reversal of drug resistance, and reprogramming of malfunction macrophages	Untraceable toxicity in mice	(128)
	Immunotherapy	H1-pAIM2/pCAIX	Exhibits the therapeutic efficacy of anti-renal carcinoma by enhancing tumor-specific multi-functional CD8 T cell responses	Low toxicity	(129)
	Chemo-photothermal combination	Ser/ICG@Lip	The high encapsulation rate of Sertraline Hydrochloride and ICG ensures the safety and therapeutic efficacy of the particle	No obvious cytotoxicity	(130)
	PTT	GSH-AuNP	GSH-AuNPs can cause higher cancer cell death when exposed to green and NIR laser light	Reducing the NP toxicity	(131)
	Chemo-sonodynamic therapy	Cur@HMON@gel	Cur@HMON accumulates in the tumor region within a biocompatible, biodegradable thermogel, avoiding drug resistance, side effects, and repeated administration of anti-tumor agents.	Low systemic toxicity	(132)
	Focused ultrasound	TKI/TSLs	Combining targeted chemotherapy, nanotechnology, and FUS shows promising potential for enhanced drug delivery and cancer treatment.	Improved RCC cytotoxicity (By MTT test)	(133)
	Drug delivery	LPs by siVEGFR2	Cav1-induced transcellular route contributes to nanoparticle accumulation in tumors	Unknown toxicity	(134)
	Targeted therapy	H1/pHGFK1	HGFK1 inhibits tumor growth, enhances anti-tumor activities of sorafenib, and reverses drug resistance in RCC	Low toxicity	(135)
	PTT	AuNRs	The combination of laser irradiation and HSA-AuNR-TKI resulted in a 100% cell kill rate	Caused around 20% cell kill	(126)
	Targeted therapy	CONP	CONPs disrupt copper transportation, induce ER stress, initiate apoptosis, and recover sunitinib responsiveness in RCC cells with sunitinib resistance	Low systemic toxicity	(136)

### Kidney cancer

Renal cancer accounted for an estimated 431,000 new cases globally in 2020, according to the World Health Organization (74) It carries a significant financial cost, which includes both direct medical expenses and indirect expenditures related to disability and loss (137). Renal cell carcinoma (RCC) is the most common type of kidney cancer, accounting for approximately 90% of all renal malignancies (138). The American Joint Committee on Cancer (AJCC) tumor-node-metastasis (TNM) staging system is the most widely utilized for RCC, where stage I and II denote localized tumors, stage III indicates regional lymph node involvement, and stage IV signifies distant metastases (139). Irrespective of disease stage, surgical resection remains the cornerstone of curative treatment for RCC in medically operable patients without contraindications (140).

#### Current treatment options and their limitations

Surgery forms the cornerstone of treatment for RCC across all stages, whenever feasible for medically eligible patients without contraindications (141). In addition to radical or partial nephrectomy, surgical removal of metastases is an option for those with stage IV disease (142). Furthermore, ablative therapies such as thermal ablation and Stereotactic Body Radiation Therapy (SBRT) are commonly employed to either shrink tumors or eliminate residual malignant cells without the need for invasive surgery (143).

In recent years, there has been widespread adoption of immunotherapy and targeted medications for advanced metastatic and recurrent RCC. Various novel agents are now standard treatments in adjuvant, metastatic, and recurrent settings, either alone or in combination therapy (144). However, it is important to note that each treatment option has its own set of drawbacks and limitations.

Surgical management as a local treatment of RCC, is a complex procedure that needs a high level of expertise, experience, and qualification. It is highly recommended to be done in high-volume centers and under the supervision of a multi-disciplinary team (145). Other than general surgical complications such as blood loss, surgical site infection, anesthesia side effects, and cardiovascular events, the surgical opening is a highly morbid incision, and removing the whole kidney results in an average 35% decrease in glomerular filtration rate (GFR) (85). Although partial excision and minimally invasive approaches are widely adopted to overcome the morbid outcomes correlated with total nephrectomy, these procedures need more expertise and often result in a higher recurrence rate due to the presence of positive surgical margins or even other malignant tumors in the remaining part of the kidney (143).

Thermal ablative techniques including Radiofrequency Ablation (RFA), Cryotherapy, and Microwave Ablation (MWA) are minimally invasive options with surgical, endoscopic, or percutaneous approaches (146). Although these methods are capable of sparing the kidney from surgical approaches, they have a learning curve and a relatively high local failure rate; Moreover, since no pathological specimen is achieved in contrast to the surgical approaches, precise histopathologic diagnosis is not possible. The imaging follow-up of these patients is also a challenge regarding the structural disruption (85, 147). SBRT is a radiation treatment indicating the use of high doses in a few fractions. Despite the ability of dose escalation to overcome the radioresistant nature of RCC being utilized as an ablative treatment in specific indications, it can result in gastrointestinal or genitourinary toxicity sometimes limiting the ultimate delivered dose (148, 149).

In systemic therapy of RCC, the main components are tyrosine kinase inhibitors (TKI) and immunotherapeutic drugs such as anti-PD-1 antibodies and anti-CTLA-4 antibodies. Chemotherapy is not generally used for RCC, except for specific subtypes in an advanced stage (150). TKIs are small molecules that inhibit the binding of adenosine triphosphate (ATP) to growth factor receptors further interrupting the intracellular cascade of phosphorylation and preventing the proliferation of malignant cells (151). Although these medications successfully prevent further tumoral proliferation in adjuvant and metastatic settings, they come with the expense of well-known adverse effects such as hypertension, diarrhea, fatigue, skin rash, and hand-foot syndrome (152).

The advent of immunotherapy dramatically changed treatment recommendations in RCC; However, as these agents alter the immune-related functions in multiple organ systems, they can result in a series of adverse effects such as inflammation in various organs and endocrine disturbances (27).

#### Nanotechnology applications in kidney cancer therapy

Nanotechnology has surfaced as a hopeful pathway for treating RCC, presenting novel methods to tackle the complexities associated with this cancer. Drug delivery methods, such as the combination of tyrosine kinase inhibitors with thermosensitive liposomes (TKI/TSL) stimulated by focused ultrasound (FUS), have shown effectiveness in enhancing drug accumulation in the tumor area and promoting regression (133). Recent findings challenge the idea that NPs may penetrate tumors through a Cav1-mediated transcellular route rather than the conventional enhanced permeability and retention (EPR) effect. Using a siRNA delivery technology (RGD-MEND), enhanced NP accumulation in vessel-rich cancers, like RCC, was observed. The study proposes a Cav1-mediated transcellular route, not the conventional EPR effect, for NP penetration into tumor vasculature. Experiments suppressing Cav1 with siRNA counteracted enhanced NP delivery, supporting the involvement of a Cav1-induced transcellular route in tumor NP accumulation (134). Exosomes, nano-sized vesicles crucial for intercellular communication, have also garnered attention, with exosomal miR-1 demonstrating significant inhibition of cell proliferation, migration, and invasion in RCC cells (153).

Exosomes, crucial for intercellular communication, involve Polymerase I and Transcript Release Factor (PTRF or CAVIN1), a potential biomarker in cancers. The study investigates the unknown mechanisms regulating exosome-related PTRF secretion. Exogenous and endogenous immunoprecipitation assays reveal UBE2O’s direct interaction and ubiquitination of PTRF. UBE2O inhibits PTRF’s impact on exosome secretion by decreasing caveolae formation. UBE2O decreases overall exosome secretion, leading to reduced PTRF release via exosomes. SDPR (CAVIN2) interacts with both UBE2O and PTRF, promoting PTRF expression in exosomes. The findings suggest that increasing UBE2O expression could be a novel approach for cancer treatment by controlling exosome-related PTRF secretion (154).

Gene therapy has also benefited from nanomedicine, with nanoparticles effectively delivering the AIM2 gene, leading to reduced cell proliferation, migration, and invasion while enhancing apoptosis in RCC cells (155). RCC lacks effective treatments, prompting interest in Absent in Melanoma 2 (AIM2) as a potential therapeutic target. AIM2 expression is significantly decreased in RCC patient specimens and renal carcinoma cell lines. Nanoparticles (H1/pAIM2) effectively deliver the AIM2 gene, increasing its expression and reducing cell proliferation, migration, and invasion while enhancing apoptosis *in vitro*. *In vivo*, intertumoral injection of H1/pAIM2 NPs inhibits tumor growth in xenograft mice. The therapeutic efficacy of H1/pAIM2 is associated with enhanced inflammasome activation, suggesting its potential as an efficient therapeutic approach for RCC treatment (155). Additionally, zinc oxide nanoparticles (ZONs) have demonstrated potential in inhibiting the RCC cell proliferation and viability by impeding lipid accumulation, oxidative stress, and reactive oxygen species (ROS) levels while upregulating miR-454-3p which targets ACSL4 (156).

Photothermal therapy (PTT) has also been explored, with an injectable thermosensitive hydrogel containing curcumin-loaded hollow mesoporous organosilica nanoparticles (Cur@HMON@gel) demonstrating sustained and controlled release of curcumin under ultrasound irradiation, generating ROS for sonodynamic tumor therapy (SDT). *In vivo* studies show that this nano-platform has high biocompatibility and biodegradability, with the potential for clinical translation in solid tumor eradication (132). Furthermore, a combination of the anticancer drug lonidamine (LND) and polydopamine (PDA) loaded onto stellate mesoporous silica nanoparticles (MSNs) and cloaked with RCC membranes (MLP@M) has exhibited enhanced synergistic effects when triggered by an 808 nm laser, improving antiproliferative and tumor-suppressing abilities for RCC treatment (157).

In the realm of image-guided surgery in RCC, extracellular vehicles (EVs) and circulating tumor cells (CTCs) have emerged as potential biomarkers, with optimized isolation protocols and microfluidic devices incorporating photovoltaic-based surface-enhanced Raman spectroscopy (SERS) platforms and shell-isolated nanoparticles (SHINs) enabling sensitive and specific identification of these biomarkers in renal cancer (158, 159). Furthermore, the RUBYchip™, a microfluidic label-free CTC detection platform, has demonstrated a remarkable efficiency of 74.9% in spiking experiments and effective clinical validation in RCC patients, positioning it as a promising tool for CTC detection in RCC management (160). By enabling sensitive and specific detection of biomarkers like EVs and CTCs, nanotechnology is providing powerful tools for image-guided surgery in RCC. These liquid biopsy approaches can reveal key information about the tumor’s presence, location, molecular characteristics, and spread - all of which can inform and guide surgical interventions and treatment strategies for optimal outcomes.

**Figure 3:**
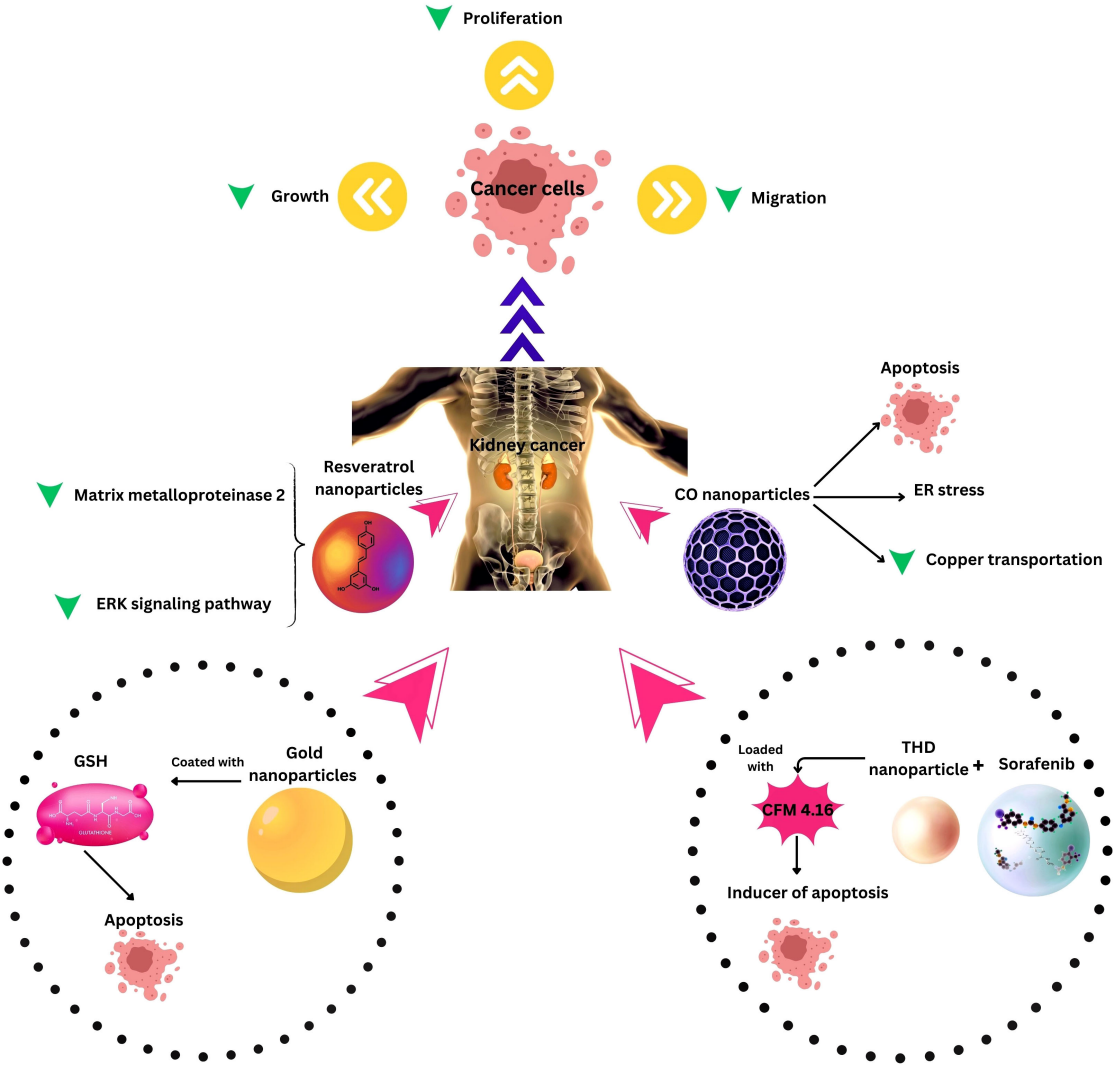
Nanotechnology applications in Kidney Cancer Therapy. ER, Endoplasmic reticulum; THD, Tumor hypoxia directed; ERK, extracellular signal-regulated kinase, CO, Cuprous oxide.

Combination therapies leveraging nanotechnology have emerged as a new approach to address the challenges associated with RCC treatment. One noteworthy development involves the investigation of long non-coding RNA SLERCC, which has demonstrated potential as a therapeutic target due to its tumor-suppressive effects. Plasmid-encapsulated nanomaterials have been explored as a novel avenue for delivering SLERCC-based therapies, offering a targeted and efficient approach to RCC treatment (161). Another breakthrough strategy involves the development of a nanoplatform that combines the tyrosine kinase inhibitor Sorafenib with a CA IX-targeted apoptosis inducer. This innovative platform effectively addresses drug resistance, a major hurdle in RCC treatment, by selectively inhibiting the growth of resistant tumors. Remarkably, this nanoplatform has demonstrated significant inhibition of resistant tumor growth, highlighting its potential as a selective and effective therapeutic approach (128). Additionally, the PH1/pHGFK1 nanoparticle has shown remarkable efficacy in inhibiting RCC growth and enhancing survival in xenografted mice models. This nanoparticle system leverages a synergistic strategy, combining multiple therapeutic modalities to achieve superior antitumor effects. The good results observed with the PH1/pHGFK1 nanoparticle underscore the potential of integrating nanotechnology with synergistic treatment approaches for more effective RCC management (135). In metastatic RCC, a combination of tyrosine kinase inhibitors and gold nanorods with photothermal ablation has showcased a synergistic effect (126), and cuprous oxide nanoparticles (CONPs) have emerged as a potential solution for sunitinib-resistant RCC by disrupting copper transportation and inducing apoptosis (136). These collective findings underscore the diverse and innovative approaches explored in RCC research, with combination therapies leveraging nanotechnology playing a pivotal role in overcoming treatment challenges and improving patient outcomes (Table and **Figure 3**).

### Less common urological cancers and current drawbacks

While prostate and bladder cancers are among the most prevalent urological malignancies, testicular, penile, and urethral cancers, though less common, can have a significant impact on patients’ lives. Testicular cancer, despite its relatively low incidence rate, is the most commonly diagnosed cancer among men aged 15-35 years (162, 163). Penile cancer, a rare malignancy, accounts for approximately 0.4-0.6% of all male cancers, but can have devastating psychological and functional consequences (164, 165). Upper tract urothelial carcinomas, while infrequent, significantly impact affected individuals due to its challenging treatment and potential consequences on quality of life (162, 166).

#### Current drawbacks in less common urological cancers

Treatment modalities, including surgery, radiation therapy, chemotherapy, and immunotherapy, aim to eradicate or control the disease. Surgical interventions, radiation therapy and chemotherapy regimens, while essential in these disease management, may cause adverse effects such as urinary incontinence, sexual dysfunction, and fatigue, further impacting patients’ physical and emotional well-being. These malignancies not only pose a threat to patients’ physical health but also carry a substantial psychosocial and economic burden, often leading to impaired quality of life, emotional distress, and financial strain (167, 168). Given the unique challenges associated with these cancers, there is an urgent need for innovative therapeutic approaches to improve patient outcomes and alleviate the burden on individuals and healthcare systems.

#### Nanotechnology applications in less common urological cancers

Nanomedicine has also shown promise in addressing the challenges associated with the treatment of less common urological cancers, including testicular, penile, and urinary tract cancers. In testicular cancer, nanoparticle-based approaches have been explored for targeted drug delivery, improving therapeutic efficacy, and reducing systemic toxicity. Hyaluronic acid (HA)-based nanoparticles have demonstrated enhanced tumor accumulation and superior antitumor activity compared to free cisplatin, a commonly used chemotherapeutic agent (139, 140). Additionally, gold nanoparticles (AuNPs) have been investigated for their ability to induce localized hyperthermia upon near-infrared (NIR) light exposure, leading to cancer cell death through photothermal therapy (PTT) (169, 170). In penile cancer, ultrasmall superparamagnetic iron oxide nanoparticles (USPIOs) have been studied in conjunction with lymphotropic nanoparticle-enhanced MRI, demonstrating higher sensitivity, specificity, and accuracy in detecting lymph node metastases compared to traditional imaging methods (171, 172). For urinary tract cancers, such as bladder cancer, novel nanomaterials like Fe3O4@PDA-VCR-FA SPs and DC-PNM-PTX have shown promising results in photothermal conversion, biocompatibility, and enhanced drug delivery (97, 173, 174). Additionally, nanoparticles like PNPs have been explored for their ability to manifest cancerous cells through heat or fluorescence under infrared light, with longer half-lives compared to conventional chemotherapeutic agents (98). Despite these advancements, further research is necessary to address challenges associated with nanoparticle-based therapies, including biodistribution, toxicity, and large-scale manufacturing, to facilitate their clinical translation for the treatment of less common urological cancers.

### Biosafety and limitations of nanomedicine application in the clinic

Nanomedicines have evolved significantly over the years and hold promise in improving early detection, accurate diagnosis, increasing treatment efficacy, and reducing side effects for urological cancers. NPs play a crucial role in the effective delivery of various therapies, including chemotherapy, gene therapy, and immunotherapy drugs, as well as in cancer diagnosis, imaging, and biomarker identification. Some notable nanoparticles that have entered human trials for urological cancers include Superparamagnetic Iron Oxide Nanoparticles (SPIONs) like Ferumoxytol, which is FDA-approved and is used as an intravenous contrast agent for MRI. Clinical studies have evaluated its utility in delineating lymph node metastasis in PCa patients, demonstrating favorable tumor detection with low toxicity (175). Albumin-bound paclitaxel (Abraxane) (176) is another nanoparticle formulation that has garnered attention, with several clinical studies indicating its ability to induce tumor response in metastatic castration-resistant prostate cancer. Notably, Abraxane achieves higher intratumoral paclitaxel concentrations and exhibits a more favorable toxicity profile compared to solvent-based paclitaxel formulations.

A comprehensive inventory profiles over 60 nanoparticle platforms explored across major treatment strategies for prostate, bladder, and renal cancers. These platforms encompass a diverse range of nanostructures, including polymeric, liposomal, gold, and magnetic formulations, designed to encapsulate and deliver chemotherapies, ablation agents, antigens, and genes. These nanoparticles enable targeted drug delivery, photothermal tumor ablation, immunostimulation, and radiosensitization, among other therapeutic modalities. The inventory highlights the favorable safety profiles of most platforms and captures the versatility of nanoparticles in addressing therapeutic gaps through tailored multifunctionality (177, 178).

Despite the promising potential of nanomedicine, the clinical translation of these innovative therapies faces several challenges. Potential toxicity concerns, limited biodistribution, and scalability issues remain significant hurdles that need to be addressed (179, 180). Rigorous preclinical and clinical testing is essential to ensure the safety and efficacy of nanomedicines, and comprehensive regulatory frameworks and guidelines are necessary for their development and approval (181). Furthermore, ongoing research is required to address the limitations and optimize the design, targeting, and delivery of nanomedicines for improved therapeutic outcomes and patient benefit (182, 183).

While significant progress has been made in the field of nanomedicine for urological cancers, further clinical translation and adoption of these innovative therapies are needed to harness their full potential and provide more effective, targeted, and personalized treatment options for patients. Collaboration between researchers, clinicians, and regulatory bodies is crucial to facilitate the successful integration of nanomedicine into clinical practice and improve patient outcomes.

## Conclusion

This comprehensive review elucidates nanotechnology’s recent strides and future prospects in confronting major clinical hurdles across common urological malignancies, such as prostate, bladder, and renal cell carcinoma. NPs have emerged as versatile platforms, offering enhanced tumor penetration, controlled drug release, targeted ligand-directed delivery to cancerous cells, and heat-triggered activation. These innovative nanoplatforms, spanning polymeric, liposomal, and metallic constructs, can revolutionize existing and novel therapeutic modalities, ranging from chemotherapy to gene therapy, thereby addressing the diverse therapeutic gaps in urological cancers.

While the inventory of nanoparticle platforms showcases promising potential and favorable safety profiles, the clinical translation of these cutting-edge technologies remains limited, with a majority of research confined to preclinical phases. To bridge this gap and facilitate widespread clinical adoption, rigorous clinical trials are imperative to establish long-term biosafety conclusively. Moreover, comparative effectiveness studies, head-to-head clinical evaluations, and cost-benefit analyses are currently lacking, underscoring the need for a comprehensive review of nanomedicine’s real-world impact.

Addressing these research imperatives through multidisciplinary collaborations among researchers, clinicians, and regulatory bodies can expedite the bench-to-bedside transition and unlock the full potential of nanomedicine in uro-oncology. The future landscape of personalized nanomedicine envisions patients with urological cancers benefiting from integrated diagnostic-therapeutic platforms, offering real-time monitoring, minimized side effects, prevention of recurrence and drug resistance, and preserving the quality of life. The advent of smart multifunctional nanostructures heralds immense promise in transforming patient outcomes across the urological cancer spectrum, ushering in a new era of targeted, effective, and personalized cancer care.

**Full Names of nanoparticles in** **Tables 1** **–** **3**:

CUR NP, Curcumin nanoparticle; AgNP-PLE, Silver nanoparticle with papaya leaf; RSV-SLN, Resveratrol with solid lipid nanoparticle; MGF-AuNP, Mangiferin functionalized gold nanoparticulate agent; PHB-PEI NP, Polyethyleneimine-functionalized polyhydroxybutyrate nanoparticle; PTX/siRNA NP-Apt, siRNAs and Paclitaxel- Aptamer-Functionalized Shell–Core nanoparticle; LNP, Lipid nanoparticle; ICGNP, Polylactide nanoparticle encapsulating indocyanine green; PEG-b-PNBMA, Polyethylene glycol)-block-poly (4,5-dimethoxy-2-nitrobenzylmethacrylate); mRNA vaccine NP, Adjuvant-pulsed mRNA vaccine nanoparticle; RALA/pDNA nanoparticle, DNA vaccination via incorporating cationic RALA nanoparticle; DTXL/GRP78 siRNA, Docetaxel/Codelivery GRP78 siRNA; GNP+ DTX, Docetaxel with Gold nanoparticle; CuSNP, Copper sulfide nanoparticle; Tf-CRC-SLN, Transferrin curcumin bioconjugated solid lipid nanoparticle; Gen@AuNP, Genistein–gold nanoparticle conjugates; @PCEC NP, PCL-based biodegradable nanoparticle; NLPs-RGD-Cur-ATO, Liposomes (arsenic trioxide/curcumin) modified with RGD peptide; Apt-PEG-siRNA@ZIF-8 nanoplatfrom, Aptamer- poly ethylene glycol- FOR Small interfering RNAs zeolitic imidazolate framework-8; Cy-KUE-OA- PSMA, Self-quenched near-infrared fluorescence probe, Cy-KUE-OA effective on Prostate-specific membrane antigen; AuNP, gold nanoparticle; PSMA-AuNP, Prostate-specific membrane antigen- gold nanoparticle; Pd-Au Nano seeds, Palladium- gold Nanoseed; PLGA NP, Poly-lactide-co-glycolic acid (PLGA) nanoparticle; Poly TTG-SS@DTX NP, Docetaxel -loaded poly-Tetraethylene glycol nanoparticle; YBS-BMS NP-RKC, Nano-photosensitizer with pH-response integrating immunogenic pyroptosis induction and immune checkpoint blockade; Au-TNF NP, Gold nanoparticle conjugated tumor necrosis factor-alpha; PNP, Paclitaxel nanoparticle; HA/CHI NP-aggregated HET, Hyaluronan/chitosan nanoparticle-aggregated heteronemin; Cat-Alg NP, Catechol-functionalized alginate (Cat-Alg) nanoparticle; Au@TNA NP, Gold -Tannic acid nanoparticle; PLZ4 NP, PLZ4 (amino acid sequence: cQDGRMGFc) nanoparticle; AB680@EMVs-aPDL1, Nanocomplexes - CD73 inhibitor (AB680)- Macrophage-derived exosome-mimetic nanovesicles (EMVs)- programmed cell death ligand 1 (aPDL1); GNP-LLO91–99, Gold nanoparticle loaded with the bacterial peptide 91–99 of the listeriolysin O toxin (GNP-LLO91–99) nanovaccines; Modified MSNP, Modified mesoporous silica nanoparticle; Nano papain, Papain nanoparticle; SYMPHONY, Synergistic Immuno Photothermal Nanotherapy; SERS NP, Surface-Enhanced Raman Scattering nanoparticle; CS/PNIPAAm@SWCNTs, (nanocomplexes) Near-Infrared Guided Thermal-Responsive Nanomedicine against Orthotopic Superficial; HG-MNS-DOX, Hydrogels- magnetic nanostructure- doxorubicin; KDM6A-mRNA, Lysine-specific demethylase 6A- mRNA; Au–Ag@PDA NP, Polydopamine-coated branched Au–Ag nanoparticle;FePPy-NH2 NP, Synthesized Fe (III)-doped polyaminopyrrole nanoparticle; UCNP-AuNR, Multifunctional nanoclusters of upconversion - and gold nanorod; Fe3O4-MNP, Fe3O4 magnetic nanoparticle; BCG-CWS, Bacillus Calmette-Guerin - cell wall skeleton; FCS Cu2-xSe NP, Fluorinated chitosan Cu2-xSe nanoparticle; TiO2, Titanium dioxide; CS/PEG NP& CS NP, Chitosan and chitosan/polyethylene glycol nanoparticle; PTX/CS NSs, Paclitaxel/chitosan nanosupensions; Sor-Mag-SLNs, Sorafenib loaded magnetic solid lipid nanoparticle; HSA-AuNR-TKI, Tyrosine kinase inhibitors and gold nanorods in human serum albumin protein; TLR7/8 agonists encapsulated in PLGA NP, TLR 7/8 bi-specific agonists encapsulated in poly(lactide-co-glycolide); CA IX-C4.16 NP, Carbonic anhydrase IX- new class of apoptosis inducer CFM 4.16 nanoparticle; H1-pAIM2/pCAIX, Folate-grafted PEI600-CyD (H1) NP-mediated DNA vaccine melanoma 2 (AIM2)/specific antigen of carbonic anhydrase IX (CAIX); Ser/ICG@Lip, Sertraline Hydrochloride and indocyanine green @Lip; GSH-AuNP, Glutathione (GSH)-modified small-sized gold nanoparticle; Cur@HMON@gel, Curcumin (Cur)-loaded hollow mesoporous organosilica NP @elastic gel matrix; TKI/TSLs, Tyrosine kinase inhibitor–loaded, thermosensitive liposomes; LPs by siVEGFR2, Long-circulating liposomes by the vascular endothelial cell growth factor receptor 2; H1/pHGFK1, First kringle domain of hepatocyte growth factor nanoparticles; CONP, Cuprous oxide nanoparticle.
