# Erratum: Clinical insights into nanomedicine and biosafety: advanced therapeutic approaches for common urological cancers

**DOI:** 10.3389/fonc.2024.1516456

**Published:** 2024-11-05

**Authors:** Frontiers Production Office

**Affiliations:** Frontiers Media SA, Lausanne, Switzerland

**Keywords:** nanomedicine, nanoparticle, urology, cancer, treatment, clinical, biosafety

Due to a production error, there was an error in affiliation 1. Instead of “School of Advanced Technologies in Medicine, Shahid Beheshti University of Medical Sciences, Tehran, Iran”, it should be “Student Research Committee, School of Advanced Technologies in Medicine, Shahid Beheshti University of Medical Sciences, Tehran, Iran”. The publisher apologizes for this mistake.

The original version of this article has been updated.

